# Supervised Machine Learning Algorithms for Bioelectromagnetics: Prediction Models and Feature Selection Techniques Using Data from Weak Radiofrequency Radiation Effect on Human and Animals Cells

**DOI:** 10.3390/ijerph17124595

**Published:** 2020-06-26

**Authors:** Malka N. Halgamuge

**Affiliations:** Department of Electrical and Electronic Engineering, The University of Melbourne, Parkville, VIC 3010, Australia; malka.nisha@unimelb.edu.au; Tel.: +61-3-8344-3933

**Keywords:** RF-EMF exposure assessment, machine learning, supervised learning, Bioelectromagnetics, human and animal cells, in-vitro studies

## Abstract

The emergence of new technologies to incorporate and analyze data with high-performance computing has expanded our capability to accurately predict any incident. Supervised Machine learning (ML) can be utilized for a fast and consistent prediction, and to obtain the underlying pattern of the data better. We develop a prediction strategy, for the first time, using supervised ML to observe the possible impact of weak radiofrequency electromagnetic field (RF-EMF) on human and animal cells without performing in-vitro laboratory experiments. We extracted laboratory experimental data from 300 peer-reviewed scientific publications (1990–2015) describing 1127 experimental case studies of human and animal cells response to RF-EMF. We used domain knowledge, Principal Component Analysis (PCA), and the Chi-squared feature selection techniques to select six optimal features for computation and cost-efficiency. We then develop grouping or clustering strategies to allocate these selected features into five different laboratory experiment scenarios. The dataset has been tested with ten different classifiers, and the outputs are estimated using the k-fold cross-validation method. The assessment of a classifier’s prediction performance is critical for assessing its suitability. Hence, a detailed comparison of the percentage of the model accuracy (PCC), Root Mean Squared Error (RMSE), precision, sensitivity (recall), 1 − specificity, Area under the ROC Curve (AUC), and precision-recall (PRC Area) for each classification method were observed. Our findings suggest that the Random Forest algorithm exceeds in all groups in terms of all performance measures and shows AUC = 0.903 where k-fold = 60. A robust correlation was observed in the specific absorption rate (SAR) with frequency and cumulative effect or exposure time with SAR×time (impact of accumulated SAR within the exposure time) of RF-EMF. In contrast, the relationship between frequency and exposure time was not significant. In future, with more experimental data, the sample size can be increased, leading to more accurate work.

## 1. Introduction

Advancing technologies that depend on wireless communication systems compel users to face increased levels of exposure to radiofrequency electromagnetic field (RF-EMF). Throughout the past decade, mobile phone use has dramatically expanded; hence, the RF-EMF exposure level to the environment has increased as a consequence [1]. This development has raised concerns on the potential hazards to human health. More than other body cells, the brain cells are vulnerable to a high specific absorption rate (SAR) because of the close proximity of the mobile phone to the users’ head in conventional usage. Hence, the potential impacts of cell phone usage on human cells, including the central nervous system (CNS) should be investigated [2]. Machine learning can be used to identify patterns of this impact and a promising way for faster, effective, and more reliable data analytics. The present study intends to investigate robust predicting techniques for identifying the impact of RF-EMF on human and animal species.

For several decades, the concerns have been elevated on the safety on long-term use of mobile phones [3,4,5,6,7,8,9,10,11,12,13,14,15,16,17,18,19,20,21,22,23,24,25,26]. The CNS is the principal concern for impacts of RF-EMF [2,27,28], as, generally, mobile phones are used in close proximity to human head [29]. The biological effects of RF-EMF exposure on human health remain vague due to inconsistent and contradictory findings of various studies [2,28].

In 2011, the World Health Organization (WHO) and the International Agency for Research on Cancer (IARC) have characterized radiofrequency radiation (RFR) originating from mobile phones as a “Possible Human Carcinogen” (Group 2B) [30] based on comprehensive in vitro, in vivo, and epidemiological studies. The Interphone Study [31] provides some evidence to imply the increased risk of glioma for heavy adult users >1640 h and the Hardell et al. study [32] shows enhanced risk of malignant brain tumors for users concerning cellular and cordless phones. In contrast, another study [33] proposes that there is no increase in risk, with several reviewing groups advising that mobile phone use is safe for adults as well as children (SCENIHR [34], ICNIRP [35]). Besides, ICNIRP [35] suggests that many experiments showed effects that neither had been independently replicated nor reproduced.

### 1.1. Background

The volume of data on the planet and around our lives appears to be ever-expanding. Big data is a phrase that defines an enormous volume of data (both structured and unstructured) that we produce on a day-to-day basis. Advanced analytic methods are performed on big data sets to extract useful information [36]. Yet, it is not the quantity but our interpretation, through the analysis of data, which is powerful and matters. Large data sets can be computationally analyzed to obtain trends, patterns, and associations. These analytics assist us in discovering what has been changed and how we should respond. For the first time, various organizations are beginning to adopt advanced analytics and, therefore, are puzzled how to utilize it.

Machine learning (ML) is the utilization of artificial intelligence (AI) [37] that produces systems with the capability to learn and enhance from experience. ML methods may operate in iterations where it attempts to discover the hidden pattern in data [38]. Discovery analytics toward big data can be facilitated by different types of analytical tools, including text analytics, data mining, statistical analysis, Structured Query Language (SQL) queries, data visualization, natural language processing, and artificial intelligence [39]. These tools have been around for quite a long time, and a considerable number of them have also been developed since the 1990s. The contrast today is that, unquestionably, more user organizations are utilizing them in association with the availability of big data. It is essential to know the analytic elements that are associated with the problem before determining which tool type is suitable for their requirements. This study aims to address new prospects for utilizing ML in Bioelectromagnetics space, allowing for the users to make intelligent judgments as they adopt it. In contrast to conventional analysis, ML mechanisms have been exploited to obtain patterns from big data that might not be feasible otherwise. Hence, algorithms can iteratively acquire hidden information from data [40].

Studying the occurrence of non-thermal biological effects of RF-EMF is crucial for distinguishing between the predictive nature of findings generated from experimental investigations in in-vitro (cell-based) and whole animals, and those arising from clinical or epidemiological studies. The impacts of past exposures and conditions can be shown in clinical or epidemiological studies. In contrast, in-vivo and in-vitro studies can be used to predict and eventually limit impacts from arising in the future [41]. Nevertheless, it cannot be expected that humans similarly react to RF-EMF as do cell cultures or animals. Numerous investigations of weak radiofrequency electromagnetic fields and radiation have concentrated on animals, plants [42,43,44], human behavioral, and cell cultures. Nevertheless, straightforward biological frameworks can contribute to our knowledge of the underlying interaction mechanisms and which proteins in living things are vulnerable to RF-EMFs. This information is essential for the advancement of the dose-response association on guidelines, as required by scientific bodies, such as the International Commission on Non-Ionizing Radiation Protection (ICNIRP) [45], IEEE, International Agency for Research on Cancer (IARC), and World Health Organisation (WHO) [1].

The production of reactive oxygen species (ROS), which is intervened by radiofrequency radiation (RFR), is considered as one of the essential bioeffect structures [46]. Mitochondria in stria marginal cells (MCs) are susceptible to ROS attack and they are meant to be very sensitive to oxidative damage [47]. A recent research finding by Yang et al. (2020) [48] into short-term exposure of mobile phone RFR, on MCs in vivo, indicates no DNA damage in marginal cells. However, the reactive oxygen species (ROS) production in the 4 W/kg exposure group was higher than that in the control group (*p* < 0.05). Various investigations [49,50,51,52] have revealed that RF-EMF exposure of animals enhances the blood-brain barrier (BBB) permeability, debilitates intracellular calcium homeostasis, changes neurotransmitters, and increments neuronal loss and harm in brain tissue.

Our recent meta-analysis [41] cross-examined published experiments that considered the non-thermal RF-EMF exposure effects (cytogenetic, gene, and protein expression analysis) on cell types with various doubling times, including lymphocytes, epithelial, endothelial, and spermatozoa from rat, mouse, and humans. Our investigation revealed that 45.3% of experiments concluded that an expansion in such potential has an effect on cells exposed to RF radiation, while 54.7% concluded that no such effects (*p* = 0.001) are observed. Nevertheless, it cannot be expected that humans similarly react to RF-EMF as do cell cultures and animals.

There is extensive clinical and epidemiological proof [41] to propose that even low degrees of radiofrequency may cause harmful consequences for the functioning of cells. Two such significant epidemiological investigations are: population-based cohorts followed for a longer time, and case-control investigations analyzing precise cases of disease and matched controls that do not have the condition [41].

**Figure 1:**
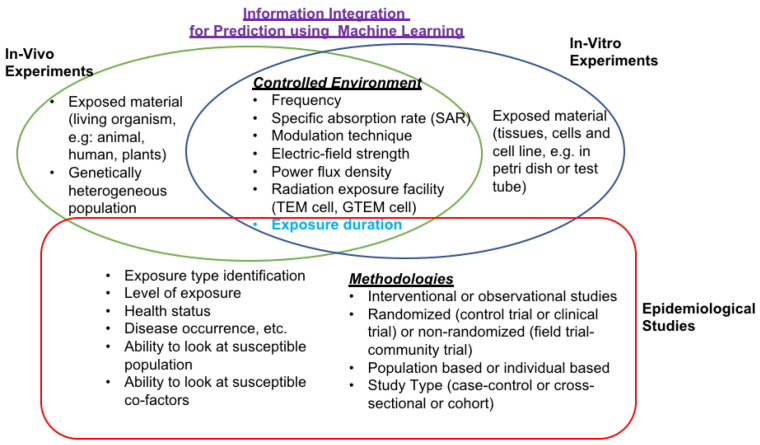
Potential features, attributes, or variables of bioelectromagnetic experiments (in-vitro, in-vivo, and epidemiological studies) that could be utilized in ML algorithms.

ML additionally improves the utilization of prediction tools to aid further health examinations (in-vitro, in-vivo, and occupational and environmental epidemiology) and allows the researchers to see how environmental properties may influence an ultimate decision. Figure 1 demonstrates the potential features or variables or attributes of bioelectromagnetic experiments (in-vitro, in-vivo, and epidemiological studies) that can be utilized by ML algorithms to predict the behavior.

### 1.2. Motivation

The advancement of emerging technology is perceived as a means to enhance and strengthen society. Advancing technologies that depend on wireless communication have begun showing higher degrees of radiofrequency electromagnetic field (RF-EMF) exposure. This enhanced the enthusiasm in the area of bioelectromagnetics, which is the examination of the impact of RF-EMF on living organisms. Currently, it is the technological era where the maturation of technology guides humans to understand the world more deeply. The insight into critical factors, which determine the impact of weak RF-EMF on living organisms, helps in a broader way to capture the underlying pattern of the data better.

The use of reliable prediction techniques to identify the effect of weak RF-EMF on organisms is turning out to be increasingly essential. An essential factor affecting the choice of algorithm is the model complexity. In classification frameworks, a model is trained and utilized to obtain predictions of an event of interest. Our previous studies used ML algorithms to predict the impact of weak RF-EMF on plant species (Table 1). This study aims to present the merit of utilizing ML algorithms (supervised learning, i.e., prediction) to develop higher accuracy classifiers for predicting the potential impact of weak RF-EMF on human and animal cells in in-vitro studies without performing in-vitro laboratory experiments. We intend to ascertain the possibility of a significant impact of the features or variables (such as frequency of weak RF-EMF, specific absorption rate (SAR), and exposure time) of weak RF-EMF exposure on human and animal cells.

**The main contributions of this paper include the following:**

1. Extract data from 300 peer-reviewed scientific publications (1990–2015) describing 1127 experimental investigations in cell-based in vitro models (human and animal species).
2. Identify the most suitable features or attributes to be utilized in prediction models to provide insight into key factors that determine the possible impact of RF-EMF in in-vitro studies while using domain knowledge, Principal Component Analysis (PCA), and Chi-squared feature selection techniques.
3. Develop a grouping or clustering strategies to allocate these selected features into five different laboratory experiment scenarios. This will produce five different feature groups or distributions for each laboratory experiment.
4. Develop a prediction model to observe the possible impact without performing in-vitro laboratory experiments. This is the first time that the supervised machine learning approach has been used for the characterization of weak RF-EMF exposure scenarios on human and animal cells.
5. Compare each classifier’s prediction performance while using seven measures to obtain the decision on its suitability, while using the percentage of the model accuracy (PCC), Root Mean Squared Error (RMSE), precision, sensitivity (recall), 1 − specificity, Area under the ROC Curve (AUC), and precision-recall (PRC Area) for each classification method.
6. Identify a robust correlation between exposure time with SAR×time (impact of accumulated SAR within the exposure period) and SAR with the frequency of weak RF-EMF on human and animal species. In contrast, the relationship between frequency and exposure time was not significant.

The rest of the paper is organized, as follows: Section 2 introduces the dataset, including its features, and how the data is collected and pre-processed, feature selection techniques, prediction models (supervised ML algorithms), features grouping strategy and evaluation measures of binary classifiers used. Subsequently, the classifier performance results are presented in Section 3 with the analysis of the prediction model and feature selection techniques carried out. Section 4 provides a related discussion. Section 5 explains potential future improvements in the area, and, finally, the paper concludes in Section 6.

## 2. Materials and Methods

In this study, nine principal classification algorithms or classifiers have been utilized, for producing accurate prediction models and observing trends of human and animal cell responsiveness to non-thermal weak RF-EMF using previously published experimental data. This study follows a few steps: data collection and preparation, optimal feature selection (attribute selection), classifier (algorithm) selection, parameter and model selection, training selected classifier, and evaluation. The ten supervised ML algorithms that were used for this analysis are (Table A1 in Appendix A): Random Forest, Bagging, J48, Decision Table, BayesNet, k-Nearest Neighbour (kNN), JRip, Support Vector Machine (SVM), Naive Bayes and Logistic Regression, and six different features (species, frequency of RF-EMF, SAR, exposure time, SAR×exposure time, and cellular response (presence or absence)). By applying dimensionally reduction techniques or feature selection methods, six major features were chosen out of all collected features. We removed two features or attributes using (i) domain knowledge, (ii) Principal Component Analysis (PCA), and (iii) the Chi-squared feature selection method. Using these techniques, we aim to gain more profound insights into the features (such as year, species, frequency of weak RF-EMF, SAR, exposure time, SAR×exposure time, and cellular response (presence or absence)) of weak RF-EMF exposure scenarios on human and animal cells. The outputs are estimated using the k-fold cross-validation method for each classifier. The most efficient classifiers have been chosen by considering the prediction accuracy and computation time.

### 2.1. Feature Selection Methods for Classification

The act of recognizing the most significant features or variables that provide the best predictive capability in modelling data is called feature selection. This is one of the key ideas in ML, which tremendously impacts the model or classifier performance. This could mean, after undergoing the feature selection process, adding or eliminating features to the model that do not enhance its performance. Features will be selected automatically or manually to provide the best to the output, or prediction features, which we are interested in. However, choosing which features we should use to build a predictive model is a challenging problem that may need require in-depth knowledge of the problem domain.

#### 2.1.1. Principal Component Analysis (PCA)

Principal Component Analysis (PCA) is an unsupervised, non-parametric statistical strategy that is predominantly utilized to reduce the dimension in a data set that consists of many features (variables or attributes) that are correlated to each other. PCA is not a classifier, and it reduces the number of features to help achieve computational and cost-efficiency. PCA does not adequately reduce data if there is a weak association between features or variables. Only when features in a dataset are highly correlated, PCA should be utilized. In contrast, using PCA is not significant if the majority of the correlation coefficients are smaller than 0.3 [55].

It is essential to normalize data before performing PCA. When the data are normalized, all of the variables produce a similar standard deviation. Consequently, all of the variables have a similar weight and PCA determines the essential ones. PCA is an approach to manage profoundly correlated variables, so that there is no compelling reason to remove those. In the event that *N* variables are profoundly correlated, then they will all place on the same Principal Component (Eigenvector) [36]. PCA is not appropriate for some classification scenarios. Assume that there are two classes of data; however, the within-class variance is high when contrasted with between-class variance; here PCA may discard the important data that isolates two classes. Consequently, if the data are noisy, and the noise variance is more than the variance between the means of the two classes, at that point, PCA will keep the noise parts, and let discard the distinctive segment (this is normal since PCA is unsupervised) [55]. In this study, we use PCA for feature selection before using ML (supervised learning) algorithms.

#### 2.1.2. Chi-Squared Feature Selection ($\chi^{2}$)

This is another filter-based strategy. In this method, the Chi-square metric between the target, and the numerical variable will be calculated and then choose the features with the maximum Chi-squared scores ($\chi^{2}$). If the number of observations in the class is close to the expected number of observations in the class then two features are independent; hence, the Chi-squared value is small. This is given by,

$$\chi^{2}=\sum_{i=1}^{n}\frac{{\left(O_{i}-E_{i}\right)}^{2}}{E_{i}}$$

where $O_{i}$ is the number of observations in the class and $E_{i}$ is the number of expected observations in class *i* [36]. The Chi-squared Ranking Filter technique is employed to determine the features that are essential for the prediction. In our analysis, we used domain knowledge, Principal Component Analysis (PCA), and Chi-squared techniques for the feature selection process.

### 2.2. Supervised Machine Learning

Machine Learning algorithms can be classified into two significant methods: supervised ML and unsupervised ML. Classification and regression methods are known as supervised ML, while clustering and association methods are known as unsupervised learning. An approach that lies in between supervised and unsupervised ML method is called semi-supervised learning.

The most practical applications utilize supervised ML algorithms (classification algorithms) for prediction. Supervised ML takes a known set of input variables, *x* (the training set), the known responses to the data or output variable (*Y*), and an algorithm that learns the mapping function or trains a model from the input to the output variables, Y=f(X). In this method, all of the data are labelled, and the algorithms attempt to figure out how to predict the output from the input data. Thus, the mapping function can be approximated adequately. With this, a classifier (ML algorithm) can predict the output variables (*Y*) for that for new input data (*x*). Since we know the outcome of the training data, we call this as supervised ML technique. The algorithm iteratively makes predictions on the training data and learning ends when the algorithm delivers a satisfactory level of performance [36].

### 2.3. Data Collection

We extracted data from 300 peer-reviewed scientific articles that were published between 1990 and 2015 that included 1127 distinct laboratory experiments to predict the potential responsiveness of human and animal cells to RF-EMF. We eliminated laboratory experiments that reported outcomes when (i) no complete dosimetry is disclosed, (ii) SAR values are greater than 50 W/kg, or (iii) exposure durations are greater than seven days and (iv) publication is not published in peer-reviewed scientific journals. Subsequently, the cellular response (presence or absence) is observed from 1127 human, rat/mouse, and other species cells. Seventy different tissue/cell types have been used to evaluate the effect of weak RF radiation from mobile phones. All of the extracted data are from peer-reviewed publications, which were published in PubMed or IEEE database.

The data employed in this analysis have been shown in our recent study (Tables 11–42, Halgamuge et al., 2020 [41]) that extracted high levels of understanding from raw data using different classification algorithms and performance evaluation methods. The collected dataset comprises of five attributes of RF-EMF and 1127 experimental case studies or instances, such as: species (human and animal cells/tissue), frequency of weak RF-EMF, SAR, exposure durations, and cellular response (presence or absence).

### 2.4. Data Pre-Processing and Inclusion Criteria

Data pre-processing was performed prior to training the supervised ML algorithms or classifiers. A portion of the data, from 300 peer-reviewed scientific publications published (1990–2015) that included 1127 distinct experimental case studies, was held as the testing part, and the remaining data were used to build classification models (training).

Data inclusion criteria and data pre-processing criteria are as shown in our previous study [53]. We initially used six features or attributes (Table 2) for the analysis; then, we used domain knowledge, PCA technique, and Chi-squared Feature Selection method to select the optimal attributes for the classifier.

Feature selection is the process of choosing features in a dataset to model the problem to be answered and understand the underlying relationships of the data. Although we had a very high data size to feature ratio (1127:6), which might not lead to overfitting on the training data, we performed the feature selection technique using (i) domain knowledge or expert knowledge, (ii) Principal Component Analysis (PCA) technique, and (iii) the Chi-squared feature selection method to select the optimal features for the classifier.

### 2.5. Data Analysis

In this work, we utilize the binary classification method that classifies the data into two groups, e.g., whether or not the non-thermal low power RF-EMF’s impact on the cellular response was observable (presence or absence). Independent variables, such as the frequency of weak RF-EMF, specific absorption rate (SAR), exposure time, and species impact on sensitive human and animal cells. A principal assumption of ML is that the training data is the representation of the distribution from which test data (future data) will be picked. The data are independent and distributed identically [36], which remains an assumption of this study. The analysis is performed using MATLAB (MathWorks Inc., Natick, MA, USA) R2019b and Weka tool (Waikato Environment for Knowledge Analysis, Version 3.9, University of Waikato, New Zealand), on a computer with macOS High Sierra (Version 10.13.6, Apple, Cupertino, CA, USA), on a computer with 1.7 GHz Intel Core i7 CPU, 4 GB 1600 MHz DDR3 RAM.

The optimal feature selection protocol is useful for identifying critical parameters that should be applied in in-vitro laboratory experiments. We used domain knowledge to select key features or attributes in our previous study [53]. However, in this study, we used domain knowledge, Principal Component Analysis (PCA) technique, and the Chi-squared feature selection method to select six optimal features for the classifier.

Cross-validation is a resampling methodology that is used to assess machine learning algorithms in a limited dataset [56]. In this work, we use the k-fold cross-validation, *k* = 10, method. Therefore, it splits the data into ten equal parts and then uses the first nine parts for training, and the final fold is for testing purposes. The cross-validation joins (averages) the proportions of fitness in prediction to determine a precise estimate of model performance.

### 2.6. Evaluation Measures of Binary Classifiers

We analyze RF-EMF sensitivity of human and animal cells while using classification algorithms. After performing the feature selection procedure, test cases were chosen to demonstrate certain aspects of the proposed method. Consequently, the k-fold cross-validation method was used to employ for each classifier. Ten classification algorithms were used to make the best predictions for the given dataset (please see Appendix A to see why each algorithm works differently). Then we analyze the correctly classified percentages of each classification algorithm.

A confusion matrix is also associated as an error matrix and is a table that is frequently used to illustrate the performance of a classifier or classification algorithm on a set of test data for when the true values are known. This provides the number of true positives TP, true negatives TN, false positives FP, and false negatives FN. We obtained the confusion matrix for each classifier, and estimated the rate of each classifier that we have utilized to predict the actual human and animal cell sensitivity and to understand if it varies using test data. The root means squared error (RMSE), mean absolute error (MAE), a weighted average of precision, recall, and F-measure are estimated using the k-fold cross-validation approach. Furthermore, correctly classified instances can be divided as TP and FP. Additionally, the incorrectly classified instances can be grouped into TN and FN. Performance evaluation measurements were used to avoid accuracy inconsistency. The confusion matrix provides a further analysis than the insignificant proportion of accuracy (correct classifications).

Binary classifiers are statistical and computational models that isolate a dataset into two groups: positives and negatives [57]. The assessment of a classifier’s prediction performance is critical to get the decision on its suitability. To date, numerous approaches have been developed and introduced to measure prediction performance. Usually, we utilize accuracy, error rate, and computation time for measuring classifier performance in terms of model development. When we consider the real performance of a classifier, accuracy is not a stable metric. If the dataset is unbalanced, accuracy will produce misleading results. Different extra measures are valuable for the assessment of the final model. Class imbalance, or a distinction in the quantities of positive and negative instances, is common in scientific areas, including the life sciences [58]. The classification of imbalanced datasets is a generally new hurdle in the field of machine learning [59]. Binary classifiers are routinely assessed while using different performance measures, for example, sensitivity and specificity, and performance is represented using Area under the Receiver Operating Characteristics (ROC) curve (AUC) plots. The ROC plots are visually attractive and they give a summary of a classifier execution over a wide scope of specificities [59]. ROC plots could be deceiving when applied in imbalanced classification situations; although, in our case, we have a balanced binary classification problem, where 45.3% indicated cell changes and 54.7% indicated no changes. The visual interpretability of ROC plots with regards to imbalanced datasets can be misjudging concerning decisions regarding the reliability of classification performance with a wrong understanding of specificity. Precision-Recall (PRC) plots, then again, can present with a precise prediction of future classification performance because of the way that they assess the portion of true positives among positive predictions [59]. Hence, in this study, we analyzed: (i) accuracy (PCC—Percent Correct Classification), (ii) error rate (RMSE), (iii) precision, *p* is the percentage of predictive items which are correct, p=TP=(TP+FP), (iv) sensitivity or recall (true positive rate), TP=(TP+FN), (v) 1− specificity (false positive rate, FP/(FP+TN), (vi) area under the ROC Curve, and (vii) precision-recall (PRC Area).

## 3. Results

Obtaining an understanding of the data is one of the goals of developing ML models. In order to predict the possible impact of RF-EMF on human and animal cells in in-vitro studies, feature selection techniques, different classifier model evaluation techniques, such as model accuracy (PCC), Root Mean Squared Error (RMSE), precision, sensitivity (recall), 1 − specificity, Area under the ROC Curve (AUC), and precision-recall (PRC Area) using the k-fold cross-validation method were used in this study. The knowledge into key components of analysis was obtained, which decide the effect of weak RF-EMF on living organisms, in order to grasp the basics of the data better, and this study is a part of it.

An overview of the utilized laboratory experiments that provided a positive association (cellular response—presence) between weak RF-EMF and for human cells (Table 3) and animal cells (Table 4).

### 3.1. Feature Selection Methods for Classification

Irrelevant or less essential features can severely affect model performance. We developed a feature selection protocol using essential domain knowledge of impact of RF-EMFs on living organism (using five different groups, as shown in Table 5). We also capture the other two approaches (Principal Component Analysis (PCA) technique and Chi-squared feature selection method) when performing feature selections techniques before utilizing in prediction models.

The SAR×exposure time is the impact of accumulated SAR within the exposure period, so we used that feature for this analysis. Finally, our analysis selects six key features (specie, frequency of weak RF-EMF, SAR, exposure time, SAR×exposure time, cellular response (presence or absence) for our dataset. Some features were removed in this analysis, for example, exposure system (GTEM cell, TEM cell, waveguide, etc.), modulation techniques of mobile communication (AM, FM, GSM, etc.), and cell line (human blood lymphocytes, breast cancer cell line, human spermatozoa, etc.).

### 3.2. Prediction Using Supervised Machine Learning

Various additional measures are useful for the evaluation of the final model. Receiver Operating Characteristics (ROC) curves can be utilized to choose the most appropriate prediction model. Hence, in this study, we utilized accuracy, error rate (RMSE), precision, sensitivity, or recall (true positive rate), 1 − specificity (false positive rate), area under the ROC Curve, and precision-recall (PRC Area).

Table 5 shows grouping or clustering strategies for allocating selected features into five groups for different laboratory experiment scenarios. First, we analyzed the accuracy of all classification algorithms for all groups, separately. The k-fold cross-validation was employed for each classifier. The Random Forest algorithm outperformed (83.56%, 0.3 s) in terms of high prediction accuracy and low computation time. Accuracy values greater than 75% are demonstrated in Table 6 (PCC>75%). We observed that the computation time was very low (less than a minute) in all algorithms for all combinations of features. Hence, the computation time for each classification algorithm was not analyzed.

**Figure 2:**
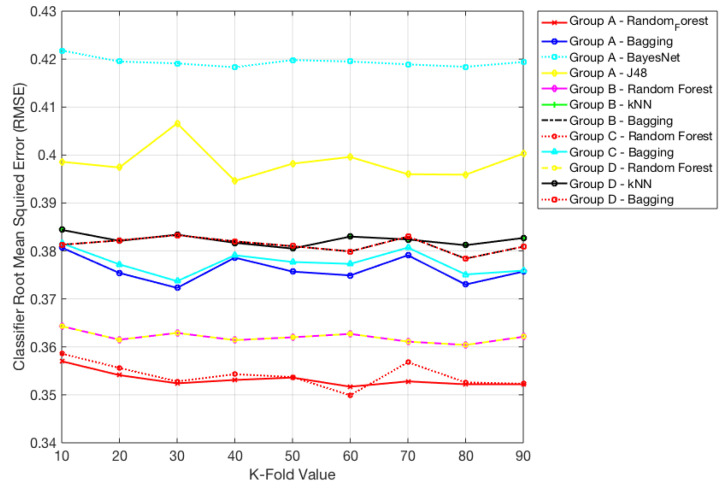
Root-mean-square error (RMSE) values <0.42 for different classifiers.

Moreover, RMSE for the best performing algorithms was plotted in Figure 2 where RMSE value <0.42.

**Figure 3:**
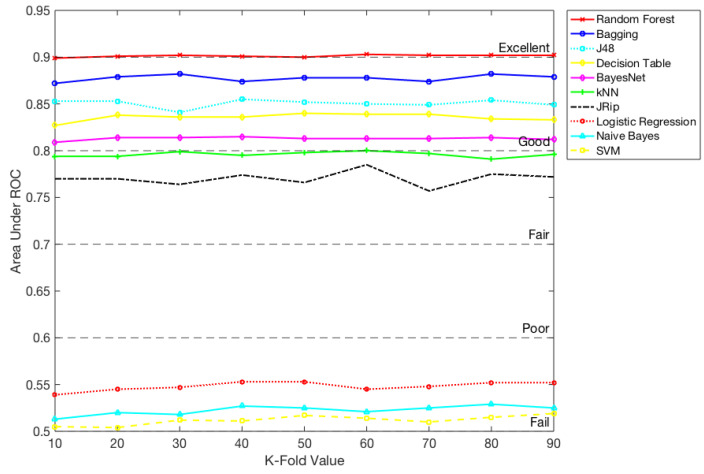
The area under the ROC Curve for all classifiers: excellent (0.9–1), good (0.8–0.9), fair (0.7–0.8), poor (0.6–0.7) and fail (0.5–0.6).

Subsequently, we analyzed Area under the ROC curve. The ROC curves are generally used to determine, graphically, the connections/trade-offs between sensitivity and specificity for every possible combination of tests. The area under the ROC Curve can be categorized based on the values: an area of 1 shows a perfect test and an area lower than 0.5 shows a worthless test. A rough guide for classifying the accuracy of a diagnostic test is the traditional academic point system is shown in Figure 3: excellent (0.9–1), good (0.8–0.9), fair (0.7–0.8), poor (0.6–0.7), and fail (0.5–0.6). This clearly demonstrates seven algorithms (Random Forest, Bagging, J48, Decision Table, BayesNet, kNN, and JRip) perform better, on the other hand, SVM, Naive Bayes, and Logistic Regression algorithms show as worthless tests, as the Area under ROC curve was less than 0.5 (Table 7). Hence, for the rest of the analysis, we only used these seven classification algorithms. The possible explanations for this result might be that each algorithm works a bit differently and each follow different computation complexities. Please see Table A1 in the Appendix A. Moreover, some of the algorithms work well in all numeric data when compared to the mixed data.

**Figure 4:**
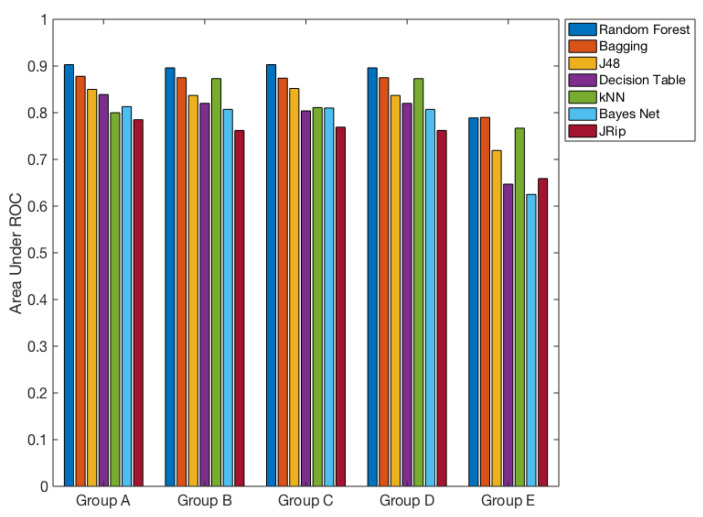
Top seven classification algorithms performed in terms of Area under the ROC Curve and accuracy out of ten algorithms that we used in this study. Group details are shown in Table 5.

We selected the top seven classification algorithms that were performed in terms of Area under the ROC Curve and accuracy (Figure 4) out of ten algorithms that we used in this study.

**Figure 5:**
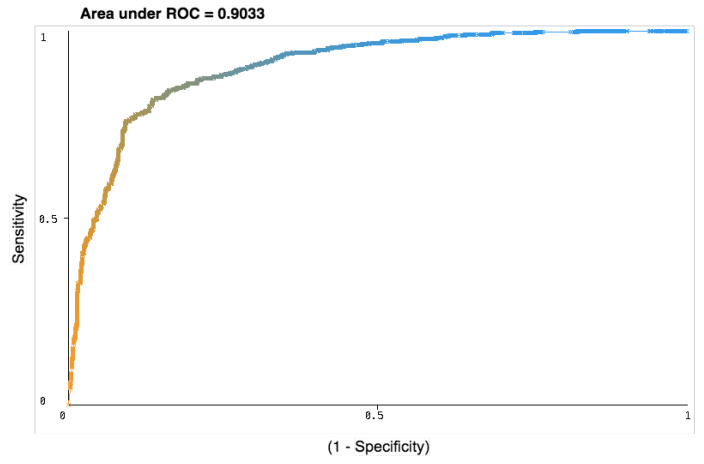
Random Forest algorithm outperforms all groups and demonstrated (AUC = 0.903 when fold = 60).

Subsequently, we estimated the classification model performance while using Group details that are shown in Table 5. This study shows negligible fluctuation with the top seven classification algorithms Area under the ROC Curve (0.93–0.8) (Figure 5 and Table 8), except Group E, demonstrating that the outcomes are crucial. Hence, this result demonstrates that the frequency of the weak RF-EMF (Hz) feature is critically important for prediction, and to better obtain the underlying pattern of the data.

Although these results reveal that the general performance of the seven classifiers, it is still interesting to know how these assessments of a classifier’s prediction performance of each algorithm are met. Hence, more importantly, the performance evaluation measures of binary classifiers are further computed while using the confusion matrix using k-fold = 60. Table 6 demonstrates the confusion matrix (weighted average) for classification model performance. Detailed comparison of the percentage of the model accuracy (PCC), Root Mean Squared Error (RMSE), precision, sensitivity (recall), 1 − specificity, Area under the ROC Curve, and precision-recall (PRC Area) for each classification method were shown here.

Precision explains how many of the positively classified instances were suitable for all algorithms or classifiers. Sensitivity (recall) shows how suitable analysis is for detecting the positives while specificity demonstrates how beneficial a test is at avoiding false alarms. Hence, all of these measures are valuable. By considering all measures, seven algorithms (Random Forest, Bagging, J48, Decision Table, BayesNet, kNN, and JRip) show high prediction performance; on the other hand, three algorithms (SVM, Naive Bayes, Logistic Regression) show unsuitability for this dataset. Computational time (CPU time) appears to be low in all classifiers due to the smaller sample size.

**Figure 6:**
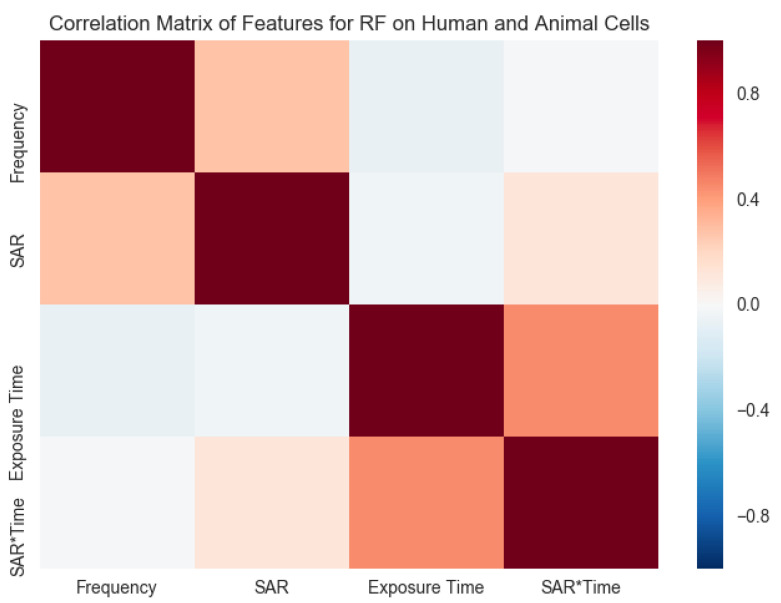
Correlations among attributes for RF-EMF on human and animal cells (maroon indicating strong correlation and blue signaling no correlation). Features that were selected for this analysis were frequency, SAR, exposure time, and SAR×exposure time (impact of accumulated SAR within the exposure period).

Figure 6 demonstrates correlations among features for RF-EMF on human and animal cells (maroon indicating strong correlation and blue signaling weak correlation). The features selected for this analysis were frequency, SAR, exposure time, and SAR×exposure time. A robust correlation was seen between exposure time with SAR×time and SAR with the frequency of weak RF-EMF. In contrast, the relationship between the frequency and exposure time was not notable. Using ML techniques, this study demonstrated more profound insights into the features of weak RF-EMF exposure scenarios on human and animal cells.

**Figure 7:**
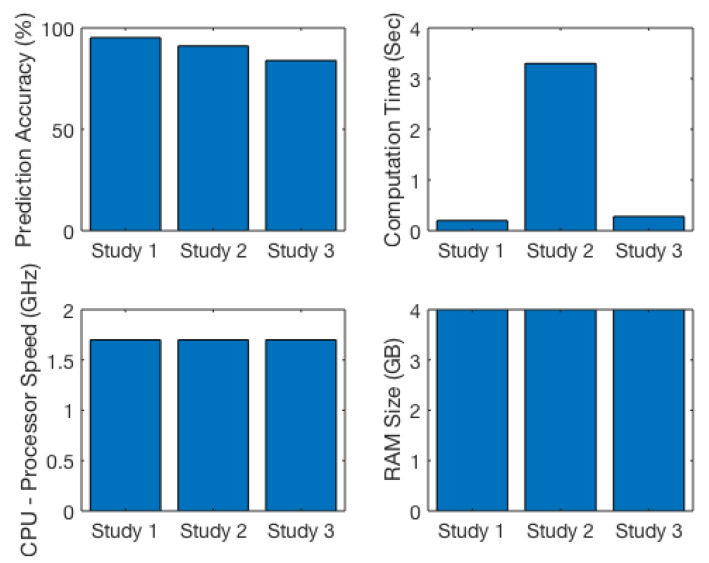
Influence of computer processor speed (CPU) and memory capacity (random-access memory (RAM) size) on prediction accuracy and computation time for Study 1, Study 2, and Study 3 (this study) shown in Table 1.

Except for the complexity of the selected algorithm, Figure 7 clearly demonstrates computation time depends on processor speed (CPU) and memory capacity (RAM size) of computer that we use to run ML algorithms. Computer with higher processor speed and RAM size provide low computation prediction time. This is essential when we use a bigger data set with more features.

## 4. Discussion

We develop up a prediction strategy to examine the possible impact of RF-EMFs on human and animal cells without performing in-vitro laboratory experiments. This is the first occasion when the supervised machine learning approach has been utilized for the characterization of weak RF-EMF exposure scenarios. In our study, we use ten different classifiers, and the outputs are estimated using the k-fold cross-validation method. The results of our study indicate that seven algorithms (Random Forest, Bagging, J48, Decision Table, BayesNet, kNN, and JRip) perform better, while SVM, Naive Bayes, Logistic Regression algorithms are shown as worthless tests, as the Area under ROC curve was less than 0.5. Our findings suggest that the Random Forest algorithm exceeds in all groups in terms of all performance measures and shows AUC = 0.903, where k-fold = 60. There are a few potential clarifications for this result. The data do not require to be re-scaled or transformed in the Random Forest method. Primarily, Random forest tackles outliers by binning them. It also handles unbalanced data. It can balance the error in class populations with unbalanced data sets. Principally, each decision tree has a high variance, though low bias. Nevertheless, since it averages all of the trees in a random forest, it also averages the variance. Hence, the Random Forest classification method has low bias and average variance model. Another possible explanation for this is that Random Forest attempts to limit the total error rate. For example, if we have an unbalanced dataset, the big class provides a low error rate, and small class provides a significant error rate. This finding also supports our previous research [53] into a prediction model that shows the Random Forest classification algorithm outperforms, with highest classification accuracy, by 95.26%.

The execution efficiency of the Random Forest algorithm increases with the number of trees. A large number of trees diminishes the danger of overfitting and variance in the model. After some point, in the Random Forest algorithm, the excess of trees can make model training inefficient by increasing the computation time [60], which results in substantial execution costs. This study does not cover memory usage for the chosen dataset. Nevertheless, a generous number of trees expends a bigger RAM space [60] when we utilize the Random Forest strategy.

We extract data from 300 peer-reviewed scientific publications (1990–2015) describing 1127 experimental investigations in cell-based in vitro models (human and animal species). A small sample was chosen because of the limitation of the in-vitro experiments that were published during the chosen period. Hidden information can be gained if we have sufficient data. ML helps to understand and verify the structure of data through mining information from data. The mechanics of learning should be automatic, as there are lots of data to be supplied by individuals themselves. Related applications (such as medical, irrigation, natural disasters) will not come from PC programs, ML specialists, or from the data itself, however, from the individuals who work with the data [36]. The utilization of data, especially data regarding individuals, has substantial ethical implications, and data mining specialists must be mindful of the ethical issues [36]. Nevertheless, when sensitive data are disposed, there is a chance that models will be built that depends on factors that can be appeared to fill in for racial or sexual attributes.

We recognize the most appropriate features or attributes to be used in prediction models to give understanding of crucial factors that decide the possible impact of RF-EMF in in-vitro studies utilizing domain knowledge, Principal Component Analysis (PCA), and Chi-squared feature selection techniques. Picking a classifier relies upon the requirements of the application. Features or attributes of classified data sets directly impact the classifier performance or the prediction rate. This is essential when using large datasets with a high number of features. We observe a very high data size to feature ratio (1127:6), which might not lead to overfitting on the training data. However, there is, in contrast to our study, a study [38] that reported a very low data size to feature ratio when predicting corn yield with ML approach.

It is becoming increasingly difficult to ignore the impact of selecting small sample sizes on prediction accuracy. Recent research by Vabalas et al. [61] has argued K-fold Cross-Validation (CV) exhibits heavily biased performance estimates with small sample sizes. Despite small sample sizes being standard, other components, which impact bias, include data dimensionality, hyper-parameter space, number of cross-validation folds, and data discriminability. For the most part, the higher the ratio of features to sample size, the higher the likelihood that a machine learning model will fit the noise in the data as opposed to the unknown underlying pattern. Additionally, the higher the quantity of adjustable parameters, the more probable that the machine learning model will overfit the data [62]. No single algorithm dominates while picking a machine learning model. Some work better with larger datasets, and some work better with the high dimensional dataset. Essentially, in this manner, it is critical to examine model viability in a specific data set.

We compare each classifier prediction performance utilizing seven measures to get the choice on its suitability, utilizing the percentage of the model accuracy (PCC), Root Mean Squared Error (RMSE), precision, sensitivity (recall), 1 − specificity, Area under the ROC Curve (AUC), and precision-recall (PRC Area) for each classification method. The assessment of a classifier’s prediction performance is essential to obtain the decision on its acceptability. Even though ROC requires exceptional care when using imbalanced datasets, it is a standard and robust measure to evaluate the performance of binary classifiers [59]. Similar to our work, previous evidence [59] suggests that precision-recall (PRC) plots can generate precise predictions of future classification performance, because of the way that they assess the portion of true positives among positive predictions.

Various correlations have been made on different classifiers executed over various datasets to find a sensible classifier for a given application. Even with high performing computers dealing with complex issues, it requires the most fitting classification algorithms to decrease the time and computation resources wastage [63]. Machine learning is an exceptional tool, since it discovers some unexplained correlations in different features in applications [53,63,64]. Nevertheless, the data type (text, numeric, images, audio, and video) [63], feature dimensions, and complexity of algorithms could impact on the performance. We build up grouping or clustering strategies to assign chosen features into five diverse laboratory experiment scenarios. This will deliver five different feature groups or distributions for every laboratory experiment. Tognola et al. found [65] cluster analysis (unsupervised learning) is a reasonable way to find features that are best at identifying the exposure situations. Supervised learning is better tailored to discover features in occupational and environmental epidemiology and public health studies [54].

More research in this space is crucial to learn whether and how some RF-EMF features (e.g., frequency of weak RF-EMF, SAR, exposure time) influence the prediction of reactions in living organisms [53]. Our previous studies used supervised ML algorithms to observe RF-EMF exposure on plants species (i) Bayes Net, NaiveBayes, Decision Table, JRip, OneR, J48, Random Tree, and Random Forest [53] and (ii) Random Forest and kNN [54]; nevertheless, this study observed performance contrasts on human and animal species. Previously developed [54] optimization technique was to characterize the trade-off among prediction accuracy and computation time based on the classification algorithm used (the Best Accuracy-Computation-time pair (BAP)). This is very vital as in many medical applications, where often prediction accuracy holds precedence over processing or computation time. In contrast, computation time is more significant in time-sensitive fields, such as natural disaster prediction.

Long-term RF-EMF exposure studies are, in general, limited in both plant and animal studies. Usually, long-term animal investigations are carried out utilizing rats and mice (both male and female) exposed for two years of RF-EMR varying between 10 and 2000 MHz, and this gives a sensible substitute to human exposure. Despite the success of short-term studies, no pathological or carcinogenic effects have been found in long-term RF-EMR studies at non-thermal levels. This includes histopathology in lifespan and hematology studies at 800 MHz, 835/847 MHz, 2450 MHz (1.3 W/kg [66] and 0.3 W/kg [67]. Nonetheless, a few pathological impacts have been published at thermal levels [68,69]. Besides, a previous study [70] has observed an increased tumor occurrence with long-term RF-EMR exposure at non-thermal levels using animals. Researchers might apply ML algorithms (supervised and unsupervised) to long-term laboratory studies utilizing whole organisms (in-vivo), and epidemiology studies to improve the accuracy of the prediction. Figure 1 shows potential features, attributes, or variables of Bioelectromagnetic experiments (in-vitro, in-vivo, and epidemiological studies) that could be utilized in ML algorithms.

Similar to animal studies, to date, there have been limited investigations exploring the long-term impacts of the RF-EMF exposure on plants, in addition to acquiring a viable conclusion on whether there is a considerable impact or not [71]. Nevertheless, there is a considerable number of short-term exposure studies demonstrate that plants have encountered physiological or morphological changes on RF-EMR (up to 13 weeks) and show statistically significant changes [71]. Conversely, the outcomes from the long-term exposure investigations demonstrate no physiological consequences for plants exposed to RF-EMR due to mobile phone radiation. This comparison of both animal and plant studies demonstrates a crucial point to the discussion on the apparent absence of long-term exposure that could interpret as, perhaps as an adaptation to RF-EMR.

Biological effects of RF-EMR from the mobile phones may depend on the frequency, mean power level and modulation of the EM signal. Numerous studies examined the health effect of the use of mobile phones. These findings are revealed from epidemiological, living organism (in vivo), and tissues in a petri dish or test tube (in vitro) studies. A lesser number of studies investigated the impacts of RF-EMF radiation on plants.

In-vitro findings are necessary to investigate natural and induced events, yet, the energies (SAR) and induced effects due to confounding elements are challenging to avoid. For example, background electromagnetic fields are non-homogenous, and temperatures inside laboratory incubators have been shown to skew results [72]. This fundamental criticism can be connected to various examinations that appear or do not exhibit biological effects. Nonetheless, organisms have in-built systems to repair the damages and maintain homeostasis [73]. The limitation of this study is the generally low sample size (1127 reported experimental case studies) to the robustness of outcomes.

Few epidemiologic studies [74,75,76,77] have associated exposure from mobile phones with neurological and cognitive dysfunctions. More repeated laboratory experiments and field studies are required [78,79,80] for future studies to additionally examine critical physical parameters that impact the biological impacts of RF-EMF. Nevertheless, the cumulative effect of mobile phone radiation is yet to be confirmed.

This study further contributes knowledge to the potential benefit of ML in the Bioelectromagnetics space. With time, a bigger sample size can be collected. Hence, further evaluations in this space are yet to be performed. We recognize a strong correlation between exposure time with SAR×time (effect of aggregated SAR within the exposure time frame) and SAR with the frequency of weak RF-EMF on human and animal species. Interestingly, the connection between frequency and exposure time was not notable. Hence, varying responses (either cellular response presence or absence) made it harder to identify [81] and measure the complex effects of weak RE-EMF. Now is the era where the progression of technology shapes how people perceive everything. Future applications in public health and occupational and environmental epidemiology should utilize ML algorithms. Additionally, the cumulative impact of weak RF-EMF demands inquiry. With time, a more significant sample size can be gathered, consequently, further assessments in this space are yet to be achieved. However, none of these findings can be directly associated with human.

## 5. Future Directions

The potential adaptability of ML algorithms in the field of Bioelectromagnetics research for human and animal cells has been explored in this study. Decision making employing predicting techniques could be the best approach. Yet, there are many factors to be investigated with regards to computation and cost-efficiency. This can be further extended by utilizing these techniques in other topics, such as in-vivo and epidemiological studies using living beings (cells, animal, plant, and human populations), as mentioned in our previous study [54]. Thorough knowledge of correlation factors between features in these studies is also essential.

### 5.1. Data, Data Size, Data Quality, Parallel, and Distributed Computing Challenges

Predicting future events by utilizing ML can be limited by poor data quality and data governance challenges. Training a classifier with poor data presents the genuine chance of producing a framework with inherent bias and unreliable or unsatisfactory results. Data researchers need to take care that the data they utilize to train their models to be as reliable and as unbiased as could be.

### 5.2. Feature Selection Strategy

Feature selection is one of the critical factors in ML, which hugely impacts model performance or classifier performance. Which features should we employ to build a predictive model is a challenging query that might need an in-depth knowledge of the problem domain? This could either mean adding features or variables to the model or removing features that do not improve model performance. Features will be chosen automatically or manually to deliver the best prediction accuracy or outputs that we prioritize. This is something to be further investigated, as predictions with more comprehensive input features is essential. In our data set, we had a very high data size to feature ratio (1127:6), which might not lead to overfitting of the training data. However, many possible future applications, such as occupational and environmental epidemiology studies, inherently provide more features in their datasets with low data size to features ratios. Hence, feature selection is an essential requirement; otherwise, built models may not hypothesize well enough to extract potentially hidden observations.

### 5.3. Machine Learning, Deep Learning, and Artificial Intelligence for Future Bioelectromagnetics

Deep learning additionally has great potential in its use in the medical field. It is “deep”, since it forms data through a wide range of layers. Hence, with a more substantial amount of data, it usually requires a high-performance computing (HPC) facility with many graphics processing units (GPUs), which are essential for calculations that are necessary for deep learning. More or less, artificial intelligence (AI) includes instructing computers to think in ways that a human might think. This is one of the emerging technologies of the modern era, and many are rushing to integrate AI with their systems. Hence, adopting AI into the bioelectromagnetics space exists as an exciting avenue to explore. The inherent adaptability of ML in the bioelectromagnetics field for human and animal cells (in-vitro) has been demonstrated and, hence, increased the likelihood that ML that could be implemented to other topics such as in-vivo and occupational and environmental studies, using animal, plant, and human populations. The still uncertain cumulative impact of weak RE-EMF demands inquiry, in-terms of laboratory experiments, in both occupational and environmental epidemiology. ML is a viable strategy for discovering features best characterizing the RF-EMF exposure scenarios; hence, it might be beneficial to better tailor occupational and environmental epidemiology and public health studies accordingly, as indicated in our previous research [54].

## 6. Conclusions

The progress of emerging technology and digital transformation are recognized to increase and intensify in the coming years. Modernized technologies that rely on wireless communication may cause increased levels of radiofrequency electromagnetic field (RF-EMF) exposure. This resulted in research interest in the space of bioelectromagnetics, which aims to investigate the consequence effect of RF-EMF on living organisms. Hence, using robust predicting methods to identify the impact has become increasingly more critical. Strong correlations were observed between SAR and exposure time of weak RF-EMF, while an insignificant relationship was observed between frequency and exposure time. As reported in our previous study (ML algorithms to predict the effect of weak RF-EMF on plants), this study (ML algorithms to predict the effect of RF-EMF on human and animal cells) also supports that the Random Forest algorithm outperforms most traditional learning algorithms in the bioelectromagnetics space. The results show that good predictive accuracy can be achieved when using feature selection methods. This study further confirmed that supervised ML is a viable strategy for discovering features best characterizing the RF-EMF exposure scenarios. Technologies are changing with time and, therefore, utilizing and recognizing the time of the study as a feature is significant. In spite of the low sample size of the study (1127 reported experimental case studies—human and animal cells in in-vitro studies) that restricted its statistical potential, this analysis demonstrates that ML algorithms can be utilized to effectively predict the impact of weak RF-EMF on human and animal cells. Feature selection is an essential strategy employing ML in bioelectromagnetics research, especially in occupational and environmental studies using animal, plant, and human populations. This is the first time that the supervised ML approach has been employed for the characterization of weak RF-EMF exposure scenarios on human and animal cells. Machine learning techniques (supervised, semi-supervised and unsupervised algorithms) contributes to innovative and practical RF-EMF exposure prediction tools. The inherent adaptability of ML in the bioelectromagnetics field for human and animal cells (in-vitro) has been demonstrated. It increases the likelihood that ML could be implemented in other areas, such as in-vivo and occupational and environmental studies, while using animal, plant, and human populations. This investigation further contributes to knowledge of the potential advantage of ML in bioelectromagnetics. This analysis may potentially improve our understanding of which features (data variables) should be gathered in the future to explain the causes of high or low weak RF-EMF exposures. In future, with more experimental data, the sample size can be increased, leading to more accurate work.

## Figures and Tables

**Table 1:** Supervised machine learning algorithms for in-vitro studies in Bioelectromagnetics: weak radiofrequency electromagnetic fields (RF-EMF) on living organisms.

Study	Experimental Type for Data Collection	Species	Data Size (No of Experimental Observations)	Features/Attributes/Variables	Machine Learning Technique	Algorithms	Prediction Accuracy (Highest)	Computation Time/CPU Time (sec)	Programming Languages, Tools and Computer Details (System Information)
**Study 01—** Halgamuge (2017) [53]	In vivo (RF-EMF directly expose to whole plants)	Plant	169	Species, frequency, SAR, power flux density, electric field strength, exposure durations, and cellular response (presence or absence)	Supervised Machine Learning (classification)	Random Forest, J48, JRip, Random Tree, Bayes Net, Naive Bayes, Decision Table, OneR	95.26%	0.2	MATLAB (MathWorks Inc., Natick, MA, USA) R2015b, one-way ANOVA procedure in SPSS Statistics (Version 23, IBM, Armonk, NY, USA) and Weka tool (Waikato Environment for Knowledge Analysis, Version 3.9, University of Waikato, Hamilton, New Zealand), on computer with 1.7 GHz Intel Core i7 CPU, 4 GB 1600 MHz DDR3 RAM
**Study 02—** Halgamuge and Davis (2019) [54]	In vivo (RF-EMF directly expose to whole plants)	Plant	169	Species, frequency, SAR, power flux density, electric field strength, exposure durations, and cellular response (presence or absence)	Supervised Machine Learning (classification)	k-Nearest Neighbor (kNN), Random Forest	91.17%	3.38–408.84	Python 3.6.0 on macOS Sierra (Version 10.12.6), on computer with 1.7 GHz Intel Core i7 CPU, 4 GB 1600 MHz DDR3 RAM
**Study 03—** Halgamuge (2020) (this study)	In-vitro (RF-EMF directly expose to human and animal cells/tissue)	Human and animal cells	1127	Species (year of study, human and animal cells/tissue), frequency, SAR, exposure durations, and cellular response (presence or absence)	Supervised Machine Learning (classification)	Random Forest, Bagging, J48, SVM (Linear Kernel), Jrip, Decision Table, BayesNet, Naive Bayes, Logistic Regression	83.56%	0.3	MATLAB (MathWorks Inc., Natick, MA, USA) R2019b and Weka tool (Waikato Environment for Knowledge Analysis, Version 3.9, University of Waikato, Hamilton, New Zealand), on a computer with macOS High Sierra (Version 10.13.6, Apple, Cupertino, CA, USA), on computer with 1.7 GHz Intel Core i7 CPU, 4 GB 1600 MHz DDR3 RAM.

**Table 2:** Descriptions of the selected six features (attributes or variables) of the analysis.

Features	Symbol	Type	Feature Type	Description (Domain)
Species (human, animal)	*c*	Nominal	Input	Different cell types have been grouped into two (human or animal cells)
Frequency of weak RF-EMF (Hz)	*f*	Numeric	Input	800–2450 (MHz)
Specific absorption rate, SAR (W/kg)	SAR	Numeric	Input	Up to 50 W/kg—Specific Absorption Rate (SAR) is a proportion of the rate at which energy is absorbed per unit mass by a living organism when exposed to a radiofrequency electromagnetic field (RF-EMF).
Duration of exposure time	T	Numeric	Input	2 min–120 h
SAR×exposure time (Halgamuge et al., 2020) [41]	$S_{T}$	Numeric	Input	Cumulative effect or impact of accumulated SAR within the exposure period
Cellular response (presence or absence)	R	Binary	Output	Presence/Absence

**Table 3:** An overview of the utilized laboratory experiments that provided positive association (cellular response—presence) between weak RF-EMF and cells.

No	Affected Cells	Frequency (Hz)	Specific Absorption Rate, SAR (W/kg)	Exposed Time (min)	Radiation Exposure Facility Details
1	Human peripheral blood mononuclear cells (PBMC)	900, 1800	0.024, 0.18, 0.4, 2, 5	15, 120, 880	Waveguide, anechoic chamber, cavity resonator
2	Human Blood Lymphocytes	800, 830, 895, 900, 905, 910, 915, 954, 1300, 1800, 1909.8, 1950, 2450	0.0054, 0.037, 0.05, 0.18, 0.21, 0.3, 0.5, 0.77, 1, 1.25, 1.5, 2, 2.5, 2.6, 2.9, 3, 3.6, 4.1, 4.3, 5, 6, 8.8, 9, 10, 12.3, 50		TEM cell, waveguide, horn antenna, wire patch cell (WPC), rectangular waveguide (R18), rectangular waveguide (WR 430), waveguide with cavity resonator, anechoic chamber with horn antenna, trumpet-like aerial
3	Human Monocytes, monocytic cells (U937), Human Mono Mac 6 cells (MM6)	900, 1300, 1800	0.18, 0.77, 1, 2, 2.5	15, 20, 60, 880	Rectangular waveguides (R18) with cavity resonator, anechoic chamber with horn antenna
4	Human B lymphoblastoid cell (TK6, CCRF-CEM)	1800	2	40, 480	Rectangular waveguides
5	Human T lymphoblastoid cells (Molt-4 T)	813.5, 836.5, 900	0.0024, 0.0026, 0.0035, 0.024, 0.026, 3.2	120, 1260, 2880	TEM cell
6	Human Leukocytes, human blood neutrophils, human white blood cells	900, 1800, 1909.8	2, 5, 10, 1909.8	15, 160, 180, 1440	TEM cell, waveguide, microstrip transmission line
7	Human leukemia cells (HL60), human erythroleukemic cells (K562)	900, 1800, 2450	0.000025, 0.000041, 1.8, 2, 2.5, 10	120, 180, 240, 360, 480, 880, 1440	GTEM cell, circular waveguide with cavity resonator, waveguide (TM01)
8	Human Whole Blood Samples, blood platelets, hemoglobin (HbA), human blood serum	835, 900, 910, 940, 2375	0.24, 0.6, 1, 1.17, 2.4, 12	1, 3, 5, 7, 15, 30, 60, 90, 120	Cavity resonator, spiral antenna setup
9	Glial cells: Astroglial (astrocytes) cells, astrocytoma cells and microglial cells	835, 900, 1800	1.8, 2.4, 2.5, 12	420, 480, 880	waveguide with cavity resonator
10	Human glioma cells (LN71, MO54, H4, SHG44)	900, 954, 2450	1.2, 1.5, 5, 10, 50	60, 120, 240, 480, 1056, 3000	GTEM cell, circular waveguide with cavity resonator
11	Human glioblastoma cells (U87MG, U251MG, A172, T98, U87)	835	2.4, 12	420	
12	Human neuroblastoma cells (NB69, SK-N-SH, SH-SY5Y, NG108-15)	872, 900, 1760, 1800, 2200	0.023, 0.086, 0.77, 1, 1.5, 1.8, 2.5, 5, 6	5, 15, 20, 30, 60, 120, 240, 480, 1440	Waveguide, wire-patch cell (WPC), waveguide with cavity resonator, chamber with a monopole antenna
13	Human primary, epidermal keratinocytes, keratinocytes cells (HaCaT)	900	2	2880	Wire-patch antenna
14	Human fibroblasts, human diploid fibroblasts, human dermal fibroblasts, human skin fibroblasts	900, 1800, 1950, 2450	0.05, 0.2, 1, 1.2, 2, 3	20, 60, 80, 320, 480, 580, 2880	Waveguide, anechoic chamber, wire-patch antenna, rectangular waveguides
15	Jurkat Cells, Jurkat human T Lymphoma cells	1800, 2450	2, 4	160, 2880	Waveguide, antenna horn
16	Embryonic carcinoma (EC-P19), Epidermoid carcinoma	1710, 1950	0.0036, 0.4, 1.5, 2	60, 120, 180, 480	Waveguide, waveguide (R14)
17	Hepatocarcinoma cell line HepG2	900, 1800, 2200	0.023, 2	20, 40, 60, 80, 1440	Waveguide, horn antena
18	Human lens epithelial cells (HLECs), eye lens epithelial cells	1800	1, 2, 3, 3.5, 4	10, 20, 30, 40, 120, 180, 480, 560, 1440	Waveguide, rectangular waveguide (R18)
19	Human epithelial amnion cells (AMA), bronchial epithelial cells (BEAS-2B), human ovarian surface epithelial cells (OSE-80PC), epithelial carcinoma cells, Human HeLa, HeLa S3	960, 1800	0.0021, 1, 2.1, 3	20, 30, 540, 3900	TEM cell, waveguide, dipole antenna
20	Human amniotic cell, amniotic epithelial cells (FL)	960, 1800	0.0002, 0.002, 0.02, 0.1, 0.5, 1, 2, 4	15, 20, 30, 40, 240	TEM cell, waveguide
21	Human breast carcinoma cells (MCF-7)	900, 1800, 2450	0.00018, 0.00036, 0.00058, 0.36, 2	60	Exposure chamber, antenna with falcon tube holder
22	Human breast epithelial cells (MCF10A), breast fibroblasts	2100	0.607	240, 1440	Horn antenna
23	Human Spermatozoa	850, 900, 1800, 1950	0.0006, 0.4, 1, 1.3, 1.46, 2, 2.8, 3, 4.3, 5.7, 10.1, 27.5	4, 10, 60, 180, 960	Waveguide, exposure chambers, omni-directional antenna, waveguide in TE10 mode with cavity resonator and monopole antenna
24	Human Endothelial cells (EA.hy926, EA.hy926v1 and EA.hy296)	900, 1800	0.77, 1.8, 2, 2.2, 2.4, 2.5, 2.8	20, 60, 480	Waveguide, exposure chamber, waveguide with resonator (TE10 mode), waveguide with cavity resonator
25	Human Trophoblast cells (HTR-8/SV neo cells)/Human lipid membrane (liposomes)	1800, 1817, 2450	0.0028, 0.0056, 2, 38	3, 10, 60, 80, 160, 320, 480	TEM cell, waveguide, dipole antenna, waveguide with cavity resonator
26	Mast cell lines (HMC-1)—mast cell leukemia	864.3	7	140	Resonant chamber
27	FC2 cells, human-hamster hybrid cells (AL)	835, 900	0.0107, 0.0172, 2	30, 120	TEM cell
28	Human adipose derived stem cells	2450	0.24	3000	
29	Human dendritic cells	1800	4	20, 240, 480	
30	Human embryonic kidney cells (HEK 293 T)	940	0.09	15, 30, 45, 60, 90	Waveguide
31	Human umbilical vein endothelial cells (HUVEC)	1800	3	20, 500	Waveguide
32	Human hair cell, human scalp hair follicle, human dermal papilla cells (hDPC)	900, 1763	0.974, 2, 10	15, 30, 60, 180, 420	Rectangular cavity-type chamber (TE102 mode)

**Table 4:** An overview of the utilized laboratory experiments that provided a positive association (cellular response—presence) between weak RF-EMF and for animal cells.

No	Affected Cells	Frequency (Hz)	Specific Absorption Rate, SAR (W/kg)	Exposed Time (min)	Radiation Exposure Facility Details
1	Rat primary microglial cells, mouse microglial cells (N9)	1800, 2450	2, 6	20, 60, 120, 240	Waveguide, rectangular horn antenna in an anechoic chamber
2	Rat glioblastoma cells (C6, C6BU-1)	1950	5.36	720, 1440, 2880	Dipole antenna
3	Rat astrocytes	872, 900, 1800, 1950	0.3, 0.46, 0.6, 1.5, 2, 2.5, 3, 5.36, 6	5, 10, 20, 60, 120, 240, 480, 520, 720, 1440, 2880, 5760	Waveguide, dipole antenna, horn antenna, rectangular waveguide
4	Rat brain capillary endothelial cells (BCEC)	1800	0.3, 0.46	2880, 5760	Rectangular waveguide
5	Mouse neuroblastoma cells (N2a, N18TG-2, NG108-15)	915	0.001, 0.005, 0.01, 0.05, 0.1	30	TEM cell
6	Rat neurons, murine cholinergic neurons (SN56)	900, 1800	0.25, 1, 2	120, 480, 1440, 2880, 4320, 5760, 7200, 8640	TEM cells, wire-patch cell, rectangular waveguides
7	Rat/mouse brain cells	1600, 2450	0.00052, 0.23, 0.48, 1.19, 1.2, 2.99, 6.42, 11.21	Cylindrical waveguide (T11 mode), cylindrical waveguide (T11 mode)
8	Rat/mouse bone marrow	2450	12	5, 10, 15	Waveguide
9	Mouse spermatozoa, Murine spermatocyte-derived cells (GC-2)	900, 1800	0.09, 1, 2, 4	20, 5040	Waveguide, rectangular waveguide
10	Embryonic mouse fibroblasts cells (C3H10T1/2, NIH3T3, L929), Mouse embryonic skin cells (M5-S), Rat1 cells	835.62, 847.74, 872, 875, 900, 915, 916, 950, 1800, 2450	0.0015, 0.024, 0.03, 0.1, 0.13, 0.24, 0.33, 0.6, 0.91, 1, 2, 2.4, 2.5, 4.4, 5	5, 10, 15, 20, 30, 40, 60, 80, 240, 480, 960, 1440, 5760	Waveguide, radial transmission line, chamber with monopole antenna, magnetron, rectangular waveguide
11	Mouse embryonic carcinoma cells (P19), Mouse embryonic stem cells, Mouse embryonic neural stem cells (BALB/c)	800, 1710, 1800	1, 1.5, 1.61, 2, 4, 5, 50	20, 60, 120	Waveguide, rectangular waveguide (R18)
12	Mouse lymphoma cells (L5178Y Tk+/-), Rat basophilic leukemia cells (RBL-2H3), Murine Cytolytic T lymphocytes (CTLL-2)	835, 915, 930, 2450	0.0081, 0.6, 1.5, 25, 40	5, 15, 30, 120, 240, 420	Waveguide, GTEM cell, anechoic chamber, aluminium exposure chamber
13	Rat granulosa cells (GFSH-R17)	1800	1.2, 2	80, 320, 480	Rectangular waveguides
14	Rat pheochromocytoma cells (PC12)	1800	2	80, 320, 480	Waveguide
15	Chinese Hamster Cells (CHO), Ovary (CHO-K1), Chinese hamster lung cells (CHL)	1800	3	20, 480	Waveguide
16	Chinese hamster fibroblast cells (V79)	864, 935, 2450	0.04, 0.08, 0.12, 0.51	15, 60, 120, 180	TEM cell, GTEM cell
17	Melanoma cell membrane (B16)	900	3.2	120	Wire patch cell (WPC)
18	Rat chemoreceptors membranes	900	0.5, 4, 12, 18	15	Waveguide (TE10 mode)
19	Hamsters pineal glands cells	1800	0.008, 0.08, 0.8, 2.7	420	Radial wave guide
20	Chick embryos	915, 2450	1.2, 1.75, 2.5, 8.4, 42.6	3, 120	TEM cell, coaxial device
21	Rabbit lens, Rabbit lens epithelial cells (RLEC)	2450	0.0026, 0.0065, 0.013, 0.026, 0.052	480	TEM cell
22	Guinea pig cardiac myocytes, pig astrocytes	900, 1300, 1800	0.001	8	TEM cell
23	Isolated frog auricle	885, 915	8, 10	10, 40	Coplanar stripline slot irradiator
24	Isolated frog nerve cord	915	20, 30		
25	Snail neurons	2450	0.0125, 0.125, 85	30, 45	Waveguide, waveguide in TE10 mode

**Table 5:** Grouping or clustering strategies to allocate these selected features into five different laboratory experiment scenarios. This will produce five different feature groups or distributions for each laboratory experiment.

Group	Selected Features
**A**	Specie, frequency of weak RF-EMF, SAR, exposure time, SAR×exposure time, cellular response (presence or absence)
**B**	Specie, frequency of weak RF-EMF, SAR, exposure time, SAR×exposure time, cellular response (presence or absence)
**C**	Frequency of weak RF-EMF, SAR, exposure time, SAR×exposure time, cellular response (presence or absence)
**D**	Specie, frequency of weak RF-EMF, exposure time, cellular response (presence or absence)
**E**	Specie, SAR, exposure time, SAR×exposure time, cellular response (presence or absence)

**Table 6:** Correctly classified instances where PCC>75% for each classification algorithm for all groups using k-fold cross-validation (Train 90% : Test 10%).

Group	Model	Fold = 10	Fold = 20	Fold = 30	Fold = 40	Fold = 50	Fold = 60	Fold = 70	Fold = 80	Fold = 90
Group A	Random Forest	82.362	82.203	83.240	83.399	82.841	83.559	83.081	83.240	83.240
Group A	kNN	76.457	76.696	76.856	76.696	76.856	77.015	76.935	76.536	76.616
Group A	Bagging	79.090	79.649	80.766	79.888	79.968	80.367	79.729	80.048	81.165
Group A	J48	78.532	78.851	78.133	79.649	78.931	78.611	79.729	79.249	78.691
Group A	Decision Table	75.579	75.658	75.419	75.020	75.738	75.579	75.977	74.940	75.179
Group B	Random Forest	80.447	81.484	80.607	81.006	80.766	81.165	81.804	81.405	80.926
Group B	kNN	79.888	80.607	80.607	80.766	80.527	80.686	80.447	80.686	80.447
Group B	Bagging	77.574	78.292	77.494	78.452	78.532	79.329	78.053	78.931	78.372
Group B	J48	75.898	78.212	77.893	78.133	77.175	78.133	78.292	78.053	77.574
Group B	Decision Table	75.658	75.339	75.738	75.339	76.297	75.818	75.818	75.579	76.058
Group C	Random Forest	82.203	82.682	82.841	83.160	82.841	83.959	83.001	83.160	83.639
Group C	kNN	78.532	78.851	78.851	79.010	79.090	79.329	79.170	78.931	78.931
Group C	Bagging	79.0902	79.569	79.809	79.489	79.888	80.048	79.649	79.569	79.729
Group C	J48	76.377	77.175	78.053	77.095	77.095	78.452	77.334	77.813	78.212
Group C	Jrip	75.020	75.579	75.578	75.499	74.860	75.499	74.940	75.419	76.217
Group D	Random Forest	80.447	81.484	80.607	81.006	80.766	81.165	81.804	81.405	80.926
Group D	kNN	79.888	80.607	80.607	80.766	80.527	80.686	80.447	80.686	80.447
Group D	Bagging	77.574	78.292	77.494	78.452	78.532	79.329	78.053	78.931	78.372
Group D	J48	75.898	78.212	77.893	78.133	77.175	78.133	78.292	78.053	77.574
Group D	Decision Table	75.658	75.339	75.738	75.339	76.297	75.818	75.818	75.579	76.056

**Table 7:** Area under the Receiver Operating Characteristics (ROC) curve (AUC) using excellent (0.9–1) and good (0.8–0.9) values in all groups (Train 90% : Test 10%).

Group	Model	Fold = 10	Fold = 20	Fold = 30	Fold = 40	Fold = 50	Fold = 60	Fold = 70	Fold = 80	Fold = 90
Group A	Random Forest	0.899	0.901	0.902	0.901	0.900	0.903	0.902	0.902	0.902
Group A	Bagging	0.872	0.879	0.882	0.874	0.878	0.878	0.874	0.882	0.879
Group A	BayesNet	0.809	0.814	0.814	0.815	0.813	0.813	0.813	0.814	0.812
Group A	J48	0.853	0.853	0.841	0.855	0.852	0.850	0.849	0.854	0.849
Group A	Decision Table	0.827	0.838	0.836	0.836	0.840	0.839	0.839	0.834	0.833
Group B	Random Forest	0.894	0.896	0.895	0.897	0.896	0.896	0.897	0.897	0.897
Group B	kNN	0.873	0.874	0.873	0.876	0.877	0.873	0.874	0.875	0.873
Group B	Bagging	0.872	0.872	0.870	0.872	0.873	0.875	0.870	0.877	0.873
Group B	BayesNet	0.807	0.810	0.810	0.810	0.808	0.807	0.806	0.808	0.807
Group B	J48	0.834	0.841	0.838	0.841	0.838	0.837	0.832	0.837	0.834
Group B	Decision Table	0.822	0.819	0.818	0.815	0.815	0.820	0.813	0.812	0.822
Group C	Random Forest	0.895	0.898	0.902	0.899	0.900	0.903	0.897	0.902	0.901
Group C	kNN	0.800	0.802	0.808	0.804	0.808	0.811	0.811	0.806	0.808
Group C	Bagging	0.870	0.876	0.881	0.874	0.876	0.874	0.872	0.88	0.878
Group C	BayesNet	0.808	0.813	0.812	0.812	0.810	0.810	0.810	0.809	0.809
Group C	J48	0.848	0.847	0.849	0.842	0.841	0.852	0.840	0.843	0.842
Group C	Decision Table	0.818	0.816	0.813	0.810	0.812	0.804	0.811	0.811	0.813
Group D	Random Forest	0.894	0.896	0.895	0.897	0.896	0.896	0.897	0.897	0.897
Group D	kNN	0.873	0.874	0.873	0.876	0.877	0.873	0.874	0.875	0.873
Group D	Bagging	0.872	0.872	0.870	0.872	0.873	0.875	0.870	0.877	0.873
Group D	BayesNet	0.807	0.810	0.810	0.810	0.808	0.807	0.806	0.808	0.807
Group D	J48	0.834	0.841	0.838	0.841	0.838	0.837	0.832	0.837	0.834
Group D	Decision Table	0.822	0.819	0.818	0.815	0.815	0.820	0.813	0.812	0.822

**Table 8:** Evaluation measures of binary classifiers: assessment of a classifier’s prediction performance where k-fold = 60 (Train 90% : Test 10%).

Classification Modle	PCC	RMSE	Precision	Sensitivity or Recall	(1− Specificity)	Area under the ROC Curve	Precision-Recall (PRC Area)
Random Forest	83.559	0.352	0.815	0.843	0.829	0.903	0.878
kNN	77.015	0.456	0.748	0.774	0.767	0.800	0.741
Bagging	80.367	0.375	0.783	0.809	0.799	0.878	0.845
SVM	52.514	0.689	0.496	0.319	0.709	0.514	0.480
Naive Bayes	51.317	0.563	0.313	0.025	0.950	0.521	0.472
Bayes Net	74.701	0.419	0.746	0.704	0.785	0.813	0.782
J48	78.611	0.399	0.752	0.816	0.759	0.850	0.803
Jrip	75.020	0.428	0.745	0.716	0.781	0.785	0.772
Decision Table	75.579	0.403	0.731	0.764	0.749	0.839	0.792
Logistic Regression	52.993	0.498	0.505	0.275	0.758	0.545	0.486

## Appendix A

**Table A1:** Supervised machine learning or classification algorithms for the analysis were used to generate the results.

Algorithm/Classifier Name	Classifier Type	Description	Capabilities (Features/Attributes Allowed by the Algorithm)	Citation
K-nearest neighbours’ classifier (kNN)	Lazy	The appropriate value of K, based on cross-validation, can be selected. The kNN (k-number of neighbours) uses the nearest neighbour search algorithm. Using cross-validation, the algorithm chooses the best k value between 1 and the value mentioned as the kNN parameter	Numeric, nominal, binary, date, unary, missing values	Aha (1991) [82]
Random Forest	Trees	Random forests algorithm builds a forest of random trees. This considers a mixture of tree predictors (where each tree depends on the independent values of a random vector sampled) and employs similar distribution for all trees in the forest. When various trees in the forest become huge, the generalization error for forests converges as far as possible to a limit. The error of the forest tree classifiers relies upon the power of the individual trees and the correlation between the trees. In this method, the data does not require to be re-scaled or transformed. Primarily, Random forest tackles outliers by binning them	Numeric, nominal, binary, date, unary, missing values	Breiman (2001) [83]
Bagging	Meta	A Bagging classifier is a meta-estimator that provides base classifiers with each on random subsets of the original dataset. Then it aggregates the prediction to form a final prediction. Such a meta-estimator can be used to reduce the variance of a black-box estimator (e.g., a decision tree) by introducing randomization into its construction procedure	Numeric, nominal, binary, date, unary, missing values	Breiman (1996) [84]
J48	Trees	The J48 is a classification algorithm which generates a decision tree which produces a pruned or unpruned C4.5 decision tree. A number of folds decide the volume of data used for reduced-error pruning. One-fold is utilized for pruning, and the rest is for growing the tree	Numeric, nominal, binary, date, unary, missing values	Quinlan (1993) [85]
Support-vector machines (SVM, Linear Kernel)	Function	The SVM classifier globally substitutes all missing values. This also transforms nominal attributes into binary values. Then, by default, it normalizes all attributes. Hence, the coefficients in the output are based on the normalized data, not the original data, which is essential for interpreting the classifier. To achieve probability estimates, use the option that provides the logistic regression method to the outputs of the support vector machine	Numeric, nominal, binary, unary, missing values	Platt (1998) [86].
Jrip	Rules	The JRip class implements a propositional rule learner called Repeated Incremental Pruning to Produce Error Reduction (RIPPER). It is established in association rules with reduced error pruning (REP), a popular and efficient method seen in decision tree algorithms. The algorithm operates through a few phases: initialization, building stage, grow phase, prune phase, optimization and selection stage	Numeric, nominal, binary, date, unary, missing values	Cohen (1995) [87]
Decision Table	Rules	The Decision Table is a class for building and utilizing an easy decision table in the classifier. It also is represented as a programming language or as in decision trees as a series of if-then-else and switch-case statements. The learning decision tables comprises choosing the correct attributes to be incorporated. A decision table is seen as balanced if it includes each conceivable mixture of input variables	Numeric, nominal, binary, date, unary, missing values	Kohavi (1995) [88].
Bayesian Network (BayesNet)	Bayes	Bayes Network is a statistical model that uses a conditional probability approach. It uses different search algorithms and quality measures. This leads to data structures (network structure and conditional probability distributions) and facilities common to Bayes Network learning algorithms. Since ADTrees are memory intensive, computer memory restrictions may arise. Nevertheless, switching this option away makes the structure learning algorithms moderate and run with more limited memory	Numeric, nominal, binary, date, unary, missing values	Friedman et al. (1997) [89].
Naive Bayes	Bayes	Naive Bayes is based on Bayes’ Theorem. It chooses numeric estimator precision values based on analysis of the training data. Due to that reason, this is not an updateable classifier which is in typical usage of initialized among zero training instances. This uses a kernel estimator for numeric attributes than a normal distribution	Numeric, nominal, binary, date, unary, missing values	John and Langley (1995) [90].
Logistic Regression	Function	Logistic regression uses a statistical technique for predicting binary classes and it estimates the probability of an event occurring. Missing values are replaced, and nominal attributes are transformed into numeric attributes using filters	Numeric, nominal, binary, date, unary, missing values	Cessie and Houwelingen [91]

## References

[1] World Health Organization (WHO) (2010). WHO Research Agenda for Radiofrequency Fields.

[2] Liu Y.X., Tai J.L., Li G.Q., Zhang Z.W., Xue J.H., Liu H.S., Zhu H., Cheng J.D., Liu Y.L., Li A.M. (2012). Exposure to 1950-MHz TD-SCDMA electromagnetic fields affects the apoptosis of astrocytes via caspase-3-dependent pathway. PLoS ONE.

[3] Frei P., Poulsen A.H., Johansen C., Olsen J.H., Steding-Jessen M., Schüz J. (2011). Use of mobile phones and risk of brain tumours: Update of Danish cohort study. BMJ.

[4] Vijayalaxmi, Cao Y., Scarfi M.R. (2014). Adaptive response in mammalian cells exposed to non-ionizing radiofrequency fields: A review and gaps in knowledge. Mutat. Res. Rev..

[5] Leszczynski D., de Pomerai D., Koczan D., Stoll D., Franke H., Albar J.P. (2012). Five years later: The current status of the use of proteomics and transcriptomics in EMF research. Proteomics.

[6] Marino C., Lagroye I., Scarfi M.R., Zenon S. (2011). Are the young more sensitive than adults to the effects of radiofrequency fields? An examination of relevant data from cellular and animal studies. Prog. Biophys. Mol. Biol..

[7] Gaestel M. (2010). Biological monitoring of non-thermal effects of mobile phone radiation: Recent approaches and challenges. Biol. Rev..

[8] Paffi A., Apollonio F., Lovisolo G.A., Marino C. (2010). Considerations for Developing an RF Exposure System: A Review for in vitro Biological Experiments. IEEE Trans. Microw. Theory Tech..

[9] McNamee J.P., Chauhan V. (2009). Radiofrequency Radiation and Gene/Protein Expression: A Review. Radiat. Res..

[10] Verschaeve L. (2009). Genetic damage in subjects exposed to radiofrequency radiation. Mutat. Res..

[11] Vijayalakshmi, Prihoda T.J. (2009). Genetic damage in mammalian somatic cells exposed to extremely low frequency electro-magnetic fields: A meta-analysis of data from 87 publications (1990–2007). Int. J. Radiat. Biol..

[12] Ruediger H.W. (2009). Genotoxic effects of radiofrequency electromagnetic fields. Pathophysiology.

[13] Vijayalaxmi, Prihoda T.J. (2008). Genetic Damage in Mammalian Somatic Cells Exposed to Radiofrequency Radiation: A Meta-analysis of Data from 63 Publications (1990–2005). Radiat. Res..

[14] Tusch H., Novak W., Molla-Djafari H. (2006). In vitro Effects of GSM Modulated Radiofrequency Fields on Human Immune Cells. Bioelectromagnetics.

[15] Verschaeve L. (2005). Genetic effects of radiofrequency radiation (RFR). Toxicol. Appl. Pharmacol..

[16] Moulder J.E., Foster K.R., Erdreich L.S. (2005). Mobile phones, mobile phone base stations and cancer: A review. Int. J. Radiat. Biol..

[17] Cotgreave I.A. (2005). Biological stress responses to radio frequency electromagnetic radiation: Are mobile phones really so (heat) shocking?. Arch. Biochem. Biophys..

[18] Vijayalaxmi, Obe G. (2004). Controversial Cytogenetic Observations in Mammalian Somatic Cells Exposed to Radiofrequency Radiation. Radiat. Res..

[19] Meltz M.L. (2003). Radiofrequency exposure and mammalian cell toxicity, genotoxicity, and transformation. Bioelectromagnetics.

[20] Ahlbom A., Juutilainen J., Veyret B., Vainio H., Kheifets L., David E. (2003). Recent Research on Mobile Telephony and Cancer and Other Selected Biological Effects.

[21] Heynick L.N., Johnston S.A., Mason P.A. (2003). Radio Frequency Electromagnetic Fields: Cancer, Mutagenesis, and Genotoxicity. Bioelectromagnetics.

[22] Matthes R. (2001). Biological Effects, Health Consequences and Standards for Pulsed Radiofrequency Fields.

[23] Brusick D., Albertini R., McRee D., Peterson D., Williams G., Hanawalt P., Preston J. (1998). Genotoxicity of radiofrequency radiation: DNA/Genetox Expert Panel. Environ. Mol. Mutagen..

[24] Verschaeve L., Maes A. (1998). Genetic, carcinogenic and teratogenic effects of radiofrequency fields. Mutat. Res./Rev. Mutat. Res..

[25] Hermann D.M., Hossmann K.A. (1997). Neurological effects of microwave exposure related to mobile communication. J. Neurol. Sci..

[26] Leonarda A., Berteaudc A.J., Bruyereb A. (1983). An evaluation of the mutagenic, carcinogenic and teratogenic potential of microwaves. Mutat. Res. Genet. Toxicol..

[27] Kim J.H., Lee J.K., Kim H.G., Kim K.B., Kim H.R. (2019). Possible Effects of Radiofrequency Electromagnetic Field Exposure on Central Nerve System. Biomol. Ther..

[28] Joubert V., Leveque P., Cueille M., Bourthoumieu S., Yardin C. (2007). No apoptosis is induced in rat cortical neurons exposed to GSM phone fields. Bioelectromagnetics.

[29] Adibzadeh F., Bakker J.F., Paulides M.M., Verhaart R.F., Van Rhoon G.C. (2015). Impact of head morphology on local brain specific absorption rate from exposure to mobile phone radiation. Bioelectromagnetics.

[30] WHO (2011). IARC Classifies Radiofrequency Electromagnetic Fields as Possibly Carcinogenic to Humans.

[31] INTERPHONE Study (2010). Brain tumour risk in relation to mobile telephone use: Results of the Interphone international case-control study. Int. J. Epidemiol..

[32] Hardell L., Carlberg M., Mild K.H. (2006). Pooled analysis of two case-control studies on use of cellular and cordless telephones and the risk for malignant brain tumours diagnosed in 1997–2003. Int. Arch. Occup. Envion. Health.

[33] Swerdlow A.J., Feychting M., Green A.C., Kheifets L., Savitz D.A. (2011). Mobile Phones, Brain Tumors, and the Interphone Study: Where Are We Now?. Environ. Health Perspect..

[34] SCENIHR (2015). Potential Health Effects of Exposure to Electromagnetic Fields (EMF).

[35] International Commission on Non-Ionizing Radiation Protection (ICNIRP) (1998). Guidelines for limiting exposure to time-varying electric, magnetic and electromagnetic fields (up to 300 GHz). Health Phys..

[36] Witten I.H., Frank E., Hall M.A., Pal C.J. (2017). Data Mining: Practical Machine Learning Tools and Techniques.

[37] Wang S., Wiart J. (2020). Sensor-Aided EMF Exposure Assessments in an Urban Environment Using Artificial Neural Networks. Int. J. Environ. Res. Public Health.

[38] Shahhosseini M., Hu G., Archontoulis S.V. (2020). Forecasting Corn Yield with Machine Learning Ensembles. arXiv.

[39] Russom P. (2011). Big Data Analytics.

[40] Kononenko I. (2001). Machine learning for medical diagnosis: History, state of the art and perspective. Artif. Intell. Med..

[41] Halgamuge M.N., Skafidas E., Davis D. (2020). A meta-analysis of in vitro exposures to weak radiofrequency radiation exposure from mobile phones (1990–2015). Environ. Res..

[42] Eberhardt J.L., Persson B.R., Brun A.E., Salford L.G., Malmgren L.O. (2008). Blood-brain barrier permeability and nerve cell damage in rat brain 14 and 28 days after exposure to microwaves from GSM mobile phones. Electromagn. Biol. Med..

[43] Halgamuge M.N., Yak S.K., Eberhardt J.L. (2015). Reduced Growth of Soybean Seedlings after Exposure to Weak Microwave Radiation from GSM 900 Mobile Phone and Base Station. Bioelectromagnetics.

[44] Sharma V.P., Singh H.P., Kohli R.K. (2009). Effect of mobile phone EMF on biochemical changes in emerging seedlings of Phaseolus aureus Roxb. Ecoscan.

[45] International Commission on Non-Ionizing Radiation Protection (ICNIRP) (2010). Guidelines for limiting exposure to time-varying electric and magnetic fields (1 Hz to 100 kHz). Health Phys..

[46] Kesari K.K., Siddiqui M.H., Meena R., Verma H.N., Kumar S. (2013). Cell phone radiation exposure on brain and associated biological systems. Indian J. Exp. Biol..

[47] Silva J., Larsson N. (2002). Manipulation of mitochondrial DNA gene expression in the mouse. Biochim. Biophys. Acta-Bioenerg..

[48] Yang H., Zhang Y., Wang Z., Zhong S., Hu G., Zuo W. (2020). The Effects of Mobile Phone Radiofrequency Radiation on Cochlear Stria Marginal Cells in Sprague-Dawley Rats. Bioelectromagnetics.

[49] Maskey D., Kim M., Aryal B., Pradhan J., Choi I.Y., Park K.S., Son T., Hong S.Y., Kim S.B., Kim H.G. (2010). Effect of 835 MHz radiofrequency radiation exposure on calcium binding proteins in the hippocampus of the mouse brain. Brain Res..

[50] Nittby H., Brun A., Eberhardt J., Malmgren L., Persson B.R., Salford L.G. (2009). Increased blood–brain barrier permeability in mammalian brain 7 days after exposure to the radiation from a GSM-900 mobile phone. Pathophysiology.

[51] Bas O., Odaci E., Mollaoglu H., Ucok K., Kaplan S. (2009). Chronic prenatal exposure to the 900 megahertz electromagnetic field induces pyramidal cell loss in the hippocampus of newborn rats. Toxicol. Ind. Health.

[52] Salford L.G., Brun A.E., Eberhardt J.L., Malmgren L., Persson B.R.R. (2003). Nerve cell damage in mammalian brain after exposure to microwaves from GSM mobile phones. Environ. Health Perspect..

[53] Halgamuge M.N. (2017). Machine Learning for Bioelectromagnetics: Prediction Model using Data of Weak Radiofrequency Radiation Effect on Plants. Int. J. Adv. Comput. Sci. Appl..

[54] Halgamuge M.N., Davis D. (2019). Lessons Learned from the Application of Machine Learning to Studies on Plant Response to Radio-Frequency. Environ. Res..

[55] Jolliffe I.T., Cadima J. (2016). Principal component analysis: A review and recent developments. Philos. Trans. A Math. Phys. Eng. Sci..

[56] Allen D.M. (1974). The Relationship between Variable Selection and Data Agumentation and a Method for Prediction. Technometrics.

[57] Tarca A.L., Carey V.J., Chen X.W., Draghici R.R.S. (2007). Machine learning and its applications to biology. PLoS Comput. Biol..

[58] Kubat M., Holte R.C., Matwin S. (1998). Machine Learning for the Detection of Oil Spills in Satellite Radar Images. Mach. Learn..

[59] Saito T., Rehmsmeier M. (2015). The Precision-Recall Plot Is More Informative than the ROC Plot When Evaluating Binary Classifiers on Imbalanced Datasets. PLoS ONE.

[60] Patel J., Shah S., Thakkar P., Kotecha K. (2015). Predicting stock and stock price index movement using trend deterministic data preparation and machine learning techniques. Expert Syst. Appl..

[61] Vabalas A., Gowen E., Poliakoff E., Casson A.J. (2019). Machine learning algorithm validation with a limited sample size. PLoS ONE.

[62] Cawley G.C., Talbot N.L. (2010). On over-fitting in model selection and subsequent selection bias in performance evaluation. Mach. Learn. Res..

[63] Singh A., Halgamuge M., Lakshmiganthan R. (2017). Impact of different data types on classifier performance of random forest, naïve bayes, and k-nearest neighbors algorithms. Int. J. Adv. Comput. Sci. Appl..

[64] Gupta A., Mohammad A., Syed A., Halgamuge M. (2016). A comparative study of classification algorithms using data mining: Crime and accidents in denver city the USA. Int. J. Adv. Comput. Sci. Appl..

[65] Tognola G., Chiaramello E., Bonato M., Magne I., Souques M., Fiocchi S., Parazzini M., Ravazzani P. (2019). Cluster Analysis of Residential Personal Exposure to ELF Magnetic Field in Children: Effect of Environmental Variables. Int. J. Environ. Res. Public Health.

[66] LaRegina M., Moros E., Pickard W., Straube W., Baty J., Roti J. (2003). The effect of chronic exposure to 835.62 MHz FDMA or 847.74 MHz CDMA radiofrequency radiation on the incidence of spontaneous tumors in rats. Radiat. Res..

[67] Frei M.R., Berger R., Dusch S., Guel V., Jauchem J., Merritt J., Stedham M. (1998). Chronic exposure of cancer-prone mice to low-level 2450 MHz radiofrequency radiation. Bioelectromagnetics.

[68] Roberts N., Michaelson S. (1983). Microwaves and neoplasia in mice: Analysis of a reported risk. Health Phys..

[69] Prausnitz S., Susskind C. (1962). Effects of chronic microwave irradiation on mice. IEEE Trans. Biomed. Electron..

[70] Chou C., Guy A., Kunz L., Johnson R., Crowley J., Krupp J. (1992). Long-term low-level microwave irradiation of rats. Bioelectromagnetics.

[71] Halgamuge M.N. (2016). Review: Weak Radiofrequency Radiation Exposure from Mobile Phone Radiation on Plants. Electromagn. Biol. Med..

[72] Portelli L., Schomay T., Barnes F. (2013). Inhomogeneous background magnetic field in biological incubators is a potential confounder for experimental variability and reproducibility. Bioelectromagnetics.

[73] Barnes F., Greenebaum B. (2016). Some effects of weak magnetic fields on biological systems: Rf fields can change radical concentrations and cancer cell growth rates. IEEE Power Electron..

[74] Foerster M., Thielens A., Joseph W., Eeftens M., Röösli M. (2018). A Prospective Cohort Study of Adolescents’ Memory Performance and Individual Brain Dose of Microwave Radiation from Wireless Communication. Environ. Health Perspect..

[75] Tyler C.R., Allan A.M. (2014). The Effects of Arsenic Exposure on Neurological and Cognitive Dysfunction in Human and Rodent Studies: A Review. Curr. Environ. Health Rep..

[76] Röösli M. (2008). Radiofrequency electromagnetic field exposure and non-specific symptoms of ill health: A systematic review. Environ. Res..

[77] Hutter H.P., Moshammer H., Wallner P., Kundi M. (2006). Subjective symptoms, sleeping problems, and cognitive performance in subjects living near mobile phone base stations. Occup. Environ. Med..

[78] Senavirathna M., Asaeda T. (2014). Radio-frequency electromagnetic radiation alters the electric potential of Myriophyllum aquaticum. Biol. Plant.

[79] Cucurachi S., Tamis W.L., Vijver M.G., Peijnenburg W.J., Bolte J.F., de Snoo G.R. (2013). A review of the ecological effects of radiofrequency electromagnetic fields (RF-EMF). Environ. Int..

[80] Halgamuge M.N. (2013). Critical Time Delay of the Pineal Melatonin Rhythm in Humans due to Weak Electromagnetic Exposure. Indian J. Biochem. Biophys..

[81] McKee L. (2018). Meeting the imperative to accelerate environmental bioelectromagnetics research. Environ. Res..

[82] Aha D., Kibler D. (1991). Instance-based learning algorithms. Mach. Learn..

[83] Breiman L. (2001). Random Forests. Mach. Learn..

[84] Breiman L. (1996). Bagging predictors. Mach. Learn..

[85] Quinlan R. (1992). C4.5: Programs for Machine Learning.

[86] Platt J. (1998). Fast Training of Support Vector Machines using Sequential Minimal Optimization. Advances in Kernel Methods—Support Vector Learning.

[87] Cohen W. Fast Effective Rule Induction. Proceedings of the Twelfth International Conference on Machine Learning.

[88] Kohavi R. The Power of Decision Tables. Proceedings of the 8th European Conference on Machine Learning.

[89] Friedman N.R., Geiger D., Goldszmidt M. (1997). Bayesian network classifiers. Mach. Learn..

[90] John G.H., Langley P. Estimating Continuous Distributions in Bayesian Classifiers. Proceedings of the Eleventh Conference on Uncertainty in Artificial Intelligence.

[91] Cessie S.L., Houwelingen J.C. (1992). Ridge Estimators in Logistic Regression. Appl. Stat..

